# Porphyrias and Mental Handicap

**Published:** 1978

**Authors:** J. Jancar

**Affiliations:** Consultant Psychiatrist, Stoke Park Group of Hospitals, Bristol and Clinical Teacher in Mental Health, University of Bristol


### Porphyrias and Mental Handicap

**J. Jancar, M.B., B.Ch., B.A.O., F.R.C.Psych., D.P.M.**
Consultant Psychiatrist, Stoke Park Group of Hospitals, Bristol and
Clinical Teacher in Mental Health, University of Bristol

The porphyrias are errors of metabolism, mainly inborn (autosomal dominant inheritance), which involve specific enzymes in the haem biosynthetic pathway. Haem biosynthesis is a function of every somatic cell.

As described by Goldberg and Rimington,[1] the history of the porphyrias began in 1841 when Scherer separated iron from dried blood and treated the iron-free residue with alcohol, which took on a blood-red colour. In 1871 Hoppe-Seyler found that the iron-free haematin was a mixture of two substances, the main one of which he called haematoporphyrin (*porphurous*- purple). Three years later Schultz published the first case of porphyria. In 1911 Günther classified the diseases of porphyrin metabolism from published cases, including his own.

Since then, numerous papers have been published and various classifications put forward. The latest classification is that from Brodie et al,[2] in which the major sites of abnormal porphyrin production are liver and bone marrow:

A **HEPATIC PORPHYRIAS**

1. Acute intermittent porphyria } Acute porphyrias
2. Variegate porphyria } Acute porphyrias
3. Hereditary coproporphyria
4. Cutaneous hepatic porphyria
   (a) Genetically predisposed
   (b) Toxic
   (c) Neoplastic

B **ERYTHROPOIETIC PORPHYRIAS**

1. Congenital porphyrias
2. Erythropoietic protoporphyria

The classifications of the porphyrias have been changing with the advancement of knowledge of the biochemistry of the disorder and with the new techniques of investigation. With the original Watson-Schwartz test many cases of porphyrias were missed or the test gave a false reading.[3] In 1965 we examined the urine of 122 epileptics with this test and found that the patients who were on promazine gave a positive reaction. An aqueous solution of promazine gave a similar reaction.[4] The various new methods of spectrometry, chromatography and the accurate measurement of porphyria concentration in urine, faeces and blood have provided the diagnosis of the types of porphyrias; recent methods of measuring enzyme levels in the blood are helping in the diagnosis of latency of this disorder.

The clinical picture of porphyrias has many facets. It was Waldenström[5] who in 1939 called porphyrias, like syphilis and hysteria, 'la petite simulatice', and Sikes[6] said in 1960: 'This disease with more faces than Eve should be kept in mind by all practitioners of the healing art, since the histories of many cases show a multitude of inaccurate diagnoses even covering periods of many years'.

Porphyrias show in the acute stage all or various combinations of signs and symptoms of colicky abdominal pain, nausea and vomiting, constipation, loss of weight, ileus, jaundice, fever, leucocytosis, peripheral neuritis, muscular atrophy, neurotic behaviour, delusions, psychosis, convulsions and skin photosensitivity. ECG abnormalities, hypertension, onycholysis, blindness, hypercholesteremia with renal impairment and hyponatremia have also been reported. The photosensitivity effect is responsible for the reddish-pink fluorescence produced by ultraviolet light in the teeth and bones of the cases of congenital porphyria, and of other tissues in cases of hepatic porphyrias.

It has been suggested that these unfortunate individuals suffering from porphyrias have given the legend of the werwolf. Their hairy faces, claw-like hands, red teeth and intermittent mental derangement have, in the primitive mind, imbued them with evil supernatural powers. The red teeth suggest a diet of blood, and photosensitivity of the skin leads to nocturnal rather than daytime sorties.[7]

Urine in the acute stage has usually a red-wine-like colour (port, burgundy or marsala) which on standing in light becomes dark mahogany-like brown.

Given at: 5th International Congress of the International Association for the Scientific Study of Mental Deficiency, Jerusalem, Israel, (1st—7th August 1979).

### DRUGS

A wide variety of drugs have been implicated in precipitating acute attacks of porphyria. Stokvis[8] first reported that an elderly woman who had taken Sulphonal, excreted a dark red urine and later died. Here is a list of some precipitating drugs[9]:

A ACUTE SYSTEMIC ATTACKS

barbiturates
sulphonamides
sulphonal
apronal (Sedormid)
phenytoin and other hydantoins
methsuximide and other succinimides
chlordiazepoxide (Librium)
dichloralphenazone (Welldorm)
meprobamate (Equanil)
carisoprodol (Carisoma)
glutethimide (Doriden)
alcohol
aminopyrine
sex hormones, various
oral contraceptives
methyldopa (Aldomet)
tolbutamide
antihistamines
griseofulvin
chloroquine
ergot preparations
imipramine (Tofranil)

B PHOTOSENSITIVITY

barbiturates alcohol sex hormones tolbutamide griseofulvin chloroquine chlorpropamide hexachlorobenzene sulphonal

**DRUGS REPORTED TO BE SAFE IN PROPHYRIAS ARE AS FOLLOWS:**

chlorpromazine chloral hydrate paraldehyde morphine pethedine methadone aspirin mefenamic acid penicillin

We have been using lithium carbonate for twelve years in one of our cases of porphyria, suffering from manic depressive psychosis, without any apparent side effects. Sodium valproate (Epilim) and bromides also appear to be safe drugs to use in porphyric patients.

### MENTAL HANDICAP

To my knowledge, there has not been a systematic study undertaken of porphyrias in mental handicap. However, a few cases have been reported in the literature. In 1962, Ackner et al,[10] reported four cases in their study of acute porphyrias in Bethlem, Maudsley and King's College Hospitals, London (three females — IQs 50, 75 and 81 and one male — IQ 70). Birchfield and Cowger[11] described a case of a borderline mentally handicapped female with epilepsy. Roth[12] reported porphyria associated with mental illness and mental handicap in a female with an IQ of 62. Porphyria in childhood following transient (30 hours) neo-natal quadriplegia was noted in a male with an IQ of 60 by Gatfield et al,[13] and hereditary coproporphyria with epilepsy in a male (IQ 34) was observed by Houston et al[14] who reported that they knew of another mentally handicapped patient who developed epilepsy at 3 years of age in whom the diagnosis of porphyria was made at the age of 29. Gregor et al[15] described a 6 month old girl with psycho-motor retardation, convulsions and bilateral congenital cataracts who suffered from acute intermittent porphyria.

I am here reporting three further cases of porphyria associated with mental handicap:

**CASE 1**

Male, 38 years of age, IQ 68, suffers from superimposed manic depressive psychosis, which has been fairly well controlled with lithium during the past twelve years.

The maternal great grandfather and paternal great grandfather were brothers. There is no other history of mental or physical illness in the family. Pregnancy was full term and normal. He is the youngest of four children. He has had otitis media from two to two and a half years of age. He attended an ordinary school where he frequently displayed bizarre and unusual behaviour. He is said to have walked and talked in his sleep. School examination revealed him to be educationally subnormal. After leaving school he was employed as a general labourer.

At the age of 16 he was admitted to Purdown Hospital (a hospital for the mentally handicapped) where he has remained since. During his stay he has suffered from severe episodes of manic depressive illness and made two suicidal attempts. Occasionally he has eczema on his legs and ears.

He was treated for his mental disorder with various anti-depressants and tranquilizers. In 1960 he was put on phenobarbitone. Soon after, he developed an acute attack of abdominal pain, vomiting, severe constipation, became very agitated, mentally deranged and suffered from insomnia. During this episode he was passing red urine which became dark on standing.

Urobilinogen was mildly increased and spectroscopic examination showed abnormal haemoglobin. Later faecal examination revealed increased coproporphyrin and protoporphyrin.

He has not had another acute attack since.

**CASE 2**

Female, 44 years old, IQ 33. Both parents are dead. Her only brother died at the age of one year. Past medical history of the family and patient is very scanty.

The patient was admitted when 14 years old to an isolation hospital with symptoms suggestive of polio-myelitis, but this was not confirmed. Since 1953 she has spent her life in various hospitals for the mentally handicapped and has been at Stoke Park Hospital since 1964. In July 1965 she had severe abdominal pains with vomiting followed by jaundice. In August 1968 she was given 400 mgs of quinalbarbitone prior to dental treatment. Next day she started to vomit, collapsed and became comatose. Gradually she responded to treatment but remained ataxic for another two days. Urine examination revealed a positive prophyrine screening test.

Her EEG shows a liability to epileptic states. She suffers from bouts of severe behaviour disorders which are treated with diazepam.

**CASE 3**

Female, 31 years old, IQ 39. A known epileptic since 8 years of age. There is no other history of mental or physical illness in the family. She has one older brother. Her mental handicap was observed at 6 years of age.

She was admitted to Stoke Park Hospital in June 1971 for two weeks' temporary care during her parents' annual holiday. Apart from her epilepsy and mental handicap it was noted that she had a mottled pigmentation of the face and she was passing a reddish dark urine. Repeated examination of urine frequently showed strong positive porphobilinogen and uroporphyrin. The faeces screening test was also positive. Electrolyte levels were within normal limits but her EEG was abnormal. Her anti-convulsive therapy on discharge was primidone and diazepam.

I recently inquired, at random, from a few hospitals for the mentally handicapped in the British Isles and I learned of five more cases of porphyria all in females:

Darenth Park Hospital, Kent
(Dr. Hatrick) — two cases
Harperbury Hospital, Herts
(Dr. Ricks) — one case
Larbert Hospital, Scotland
(Dr. Primrose) — one case
Monyhull Hospital, Birmingham
(Dr. Liu) — one case

From the cases in this survey there are 13 females, 4 males and one of unspecified sex from which a prevalence of females suffering from porphyria is apparent.

It is also interesting to note that most of the reported cases, including ours, of porphyrias associated with mental handicap had clinical signs and symptoms of this disorder in early childhood or adolescence.

No doubt I have missed other reported cases in the world literature and there must be more cases among the mental handicap population awaiting diagnosis.

### DISCUSSION

The book 'Diseases of Porphyria Metabolism' by Goldberg and Rimington[1] and the great detective story 'The Porphyrias — a story of inheritance and environment' by Dean[16] aroused great interest in this disorder. The publication about porphyria in our own Royal Family[17] created further world-wide interest and controversy as did the study 'The Porphyria of Heinrich Heine'.[18]

Since then a number of surveys have been made in various branches of medicine, including psychiatry. It has been reported that between 50—75% of people suffering from acute attacks of porphyria, who include children, adults and mentally handicapped, suffer from psychiatric disorders.[19] Forensic psychiatrists have reported cases of porphyria.[20] The incidence of fits complicating an acute attack of porphyria in adults, according to Goldberg and Rimington,[1] is about 15%, but the association of chronic epilepsy with porphyria is not yet well documented. However, if epilepsy is getting worse with anticonvulsive therapy, particularly with the drugs mentioned before which precipitate porphyria, the investigation for porphyria is imperative.

*continued on page 11*

*continued from page 5*

Vigilance over new drugs and new chemical compounds which may cause porphyria is very important. An example of this is the Turkish epidemic of porphyria which took place in 1954 after seed wheat had ben treated with the fungicide hexachlorobenzene. Over 5000 people were affected, of whom about 4500 were children under 16 years, with a 10% mortality rate (2—5 years 95%).

Inheritance of congenital porphyria in bovines and pigs has also been reported and experimental porphyria were produced with various drugs in dogs, rabbits, rats and fowls.[1]

Two world congresses on porphyria, one in 1963 (Cape Town) and another in 1975 (Freiburg), contributed greatly towards the diagnosis of porphyria and produced further biochemical evidence regarding its aetiology and pathology.

### TREATMENT

Modern intensive care has done much to lessen the mortality from attacks of acute porphyria — from 9%

to 30% for a series of patients collected over the past two decades — but progress towards an effective, specific treatment has been slow.

Carbohydrate loading is now widely used in the management of acute attacks of porphyria.

Prompt and often dramatic recoveries following haematin infusions have been reported. As with carbohydrate infusions, objective assessment of the effectiveness of haematin infusion is difficult and, promising as it seems, this form of treatment is still under evaluation.

Prevention of the acute attacks remains as important as ever. This approach which is based on diagnosis during the symptomless phase and avoidance of known precipitants has been strengthened by the introduction of erythrocyte uroporphyrinogen—1—synthase measurements for the diagnosis of acute intermittent porphyria and coproporphyrinogen oxidase measurements for hereditary coproporphyria.[21]

### CONCLUSION

During the past twenty years great advances have been made regarding diagnosis, classifications and prevention of porphyrias. No doubt further clinical, biochemical and genetic studies will eventually curtail the activities of this elusive 'purple pimpernel' and its disguises.

I wish to conclude this short paper with the last four lines from Dean's book 'The Porphyrias' — 'It has involved the study of the formation of a people, and has been an exciting voyage of discovery which still continues the everlasting whisper — something hidden, go and find it'.

### ACKNOWLEDGEMENTS

I wish to thank Dr. R. D. Eastham, and his staff, Frenchay Hospital, Bristol, for the biochemical estimations and Miss Anita England for secretarial work.

### REFERENCES

1. GOLDBERG, A. and RIMINGTON, C. (1962). *Diseases of Porphyrin Metabolism.* Charles C. Thomas Springfield, Ill.
2. BRODIE, M. J., MOORE, M. R. and GOLDBERG, A. (1977). Enzyme Abnormalities in the Porphyrias. *Lancet,* **II**, 699—701.
3. OTTOSSON, J.-O. and PERRIS, C. (1971). Screening for Porphyria among Psychiatric Patients. *Acta Psychiatrica Scandinavica* — Supplementum 221 — Munksgaard Copenhagen, 128—132.
4. JANCAR, J. and PHILPOT, G. R. (1965). Porphobilinogen-like Chromogens in Urine of Epileptics. *British Medical Journal,* **II**, 1498.
5. WALDENSTRÖM, J. (1939). Neurological Symptoms Caused by So-called Acute Porphyria. *Acta psychiatrica et neurologica,* **14**, 375—379.
6. SIKES, Z. S. (1960). Electroencephalographic Abnormalities and Psychiatric Manifestations in Intermittent Porphyria. *Diseases of the Nervous System,* **21**, 226—229.
7. ILLIS, L. (1964). On Porphyria and the Aetiology of Werwolves. *Proceedings of the Royal Society of Medicine,* **57**, 23—26.
8. STOKVIS, B. J. (1889). (quoted by Goldberg and Rimington, 1962).
9. BARNETT, I. G. (1971). Porphyria Varieguta Presenting as Postpartum Hypertension and Epilepsy. *Proceedings of the Royal Society of Medicine,* **64**, 34—46.
10. ACKNER, B., COOPER, J. E., GRAY, C. H. and KELLY, MARGARET (1962). Acute Porphyria: A Neuropsychiatric and Biochemical Study. *Journal of Psychosomatic Research,* **6**, 1—24.
11. BIRCHFIELD, R. I. and COWGER, MARILYN L. (1966). Acute Intermittent Porphyria with Seizures. Anticonvulsant Medication-Induced Metabolic Changes. *American Journal of Diseases of Children,* **112**, 561—565.
12. ROTH, N. (1968). The Psychiatric Syndromes of Porphyria. *International Journal of Neuropsychiatry,* **4**, 32—44.
13. GATFIELD, P. D., HAUST, H. L. and DURRANT, D. (1972). Porphyria in Childhood Following Transient Neo-natal Quadriplegia. *Developmental Medicine and Child Neurology,* **14**, 495—501.
14. HOUSTON, A. B., BRODIE, M. J., MOORE, M. R., THOMPSON, G. G. and STEPHENSON, J. B. P. (1977). Hereditary Coproporphyria and Epilepsy. *Archives of Disease in Childhood,* **52**, 646—650.
15. GREGOR, ANITA, KOSTRZEWSKA, EWA, PROKURAT, HALINA, PUCEK, ZOFIA and TORBICKA, EMILIA (1977). Increased Protoporphyrin in Erythrocytes in a Child with Acute Intermittent Porphyria. *Archives of Disease in Childhood,* **52**, 947—50.
16. DEAN, G. K. (1963). *The Porphyrias: A Story of Inheritance and Environment.* Pitman Medical, London. (Second Edition, 1971).
17. MACALPINE, IDA, HUNTER, R. and RIMINGTON, C. (1968). Porphyria in the Royal Houses of Stuart, Hanover and Prussia. A Follow-up Study of George III's Illness. *British Medical Journal,* **I**, 7—18.
18. ROTH, N. (1969). The Porphyria of Heinrich Heine. *Comprehensive Psychiatry,* **10**, 90—106.
19. BARCLAY, N. (1974). Acute Intermittent Porphyria in Childhood — A Neglected Diagnosis? *Archives of Disease in Childhood,* **49**, 404—406.
20. TRAFFORD, P. A. (1976). Homicide in Acute Porphyria. *Forensic Science,* **7**, 113—120.
21. *Lancet* (1978). Treatment of Acute Hepatic Porphyria, **I**, 1024—1026.

